# A 2x folic acid treatment affects epigenetics and dendritic spine densities in SHSY5Y cells

**DOI:** 10.1016/j.bbrep.2019.100681

**Published:** 2019-08-17

**Authors:** Rahaf Al Sayed, Whitnei Smith, Nicole Rogers, Nuri Smith, Daniel Clark, Gabriel Castillo, Hunter McLeod, Stewart Glenister, Kimberly R. Shorter

**Affiliations:** University of South Carolina Upstate Division of Natural Sciences and Engineering, 800 University Way, Spartanburg, SC, 29303, USA

**Keywords:** Epigenetics, Folic acid, Dendritic spines, DNA methylation, Gene expression, Histone modifications, ASD, Autism Spectrum Disorders, DNMT, DNA Methyltransferase, FA, Folic Acid, HAT, Histone Acetyltransferase, HDAC, Histone Deacetylase, HMT, Histone Methyltransferase, MBD2, Methyl-CpG Binding Domain Protein 2, MECP2, Methyl-CpG Binding Protein 2

## Abstract

Many diseases are now associated with aberrant epigenetics and gene expression changes. Epigenetics can be modified by factors like diet. One dietary factor, folic acid, is consumed in various forms including supplements, energy drinks, and fortified grains. It was hypothesized high levels of folic acid would affect gene expression and enzyme activity of chromatin modifying enzymes as well as dendritic spine densities in a commonly utilized neuron model, the SHSY5Y cell. Decreased MBD2 and MECP2 were discovered upon treatment of SHSY5Y cells with a 2x folic acid dose. Corresponding decreases in dendritic spines were apparent in the 2x folic acid treated cells as well. Activity of DNMTs and H3K4 HMTs was altered. Further, H3K4me1, H3K4me3, H3K9Ac, and global DNA methylation were decreased in the 2x folic acid treated cells. Further studies are warranted to determine if the effects of excess folic acid are detrimental to organismal physiology.

## Highlights

• The 2x folic acid treatment in SHSY5Y cells induces a significant decrease in MBD2 and MECP2 mRNA expression.
• The excess folic acid also causes a significant increase in dendritic spines in SHSY5Y cells.
• The 2x folic acid treatment increases DNMT activity and significantly decreases H3K4 HMT activity in SHSY5Y cells.
• These findings align with some of those seen in autism spectrum disorders.

## 1 Introduction

Epigenetic modifications, including DNA methylation and histone methylation and acetylation marks, have received great attention recently in various research fields since altered epigenetics is associated with various diseases including neurodevelopmental disease, psychiatric illness, and cancer [1,2]. DNA methylation is usually associated with decreased gene expression. However, histone methylation affects gene expression in a residue-specific manner, where the location and type of methylation mark influences whether there is gene silencing. Histone acetylation generally is associated with open chromatin—or euchromatin—and a subsequent increase in gene expression.

Several factors influence epigenetic modifications, including stress and diet. One dietary factor, folic acid (FA), contributes to the one-carbon metabolic pathway where a methyl group is formed. The methyl group is added to either DNA using DNA Methyltransferases (DNMTs) or to histones using Histone Methyltransferases (HMTs). Notably, histone methylation affects histone acetylation [3], which relies on functioning of histone acetyltransferases (HATs) and/or histone deacetylases (HDACs). For example, an increase in H3K4me3 (histone 3, lysine 4 trimethylation) increases H3K9Ac (histone 3, lysine 9 acetylation) [3] indicating an effect of histone methylation on HAT and/or HDAC expression or function. Therefore, FA may play a significant role in regulating levels of various epigenetic marks.

With the potential to alter gene expression on a large scale, FA overconsumption has been a target of various studies. Many generally consider FA overconsumption harmless, but over-exposure of humans to FA fortification led to higher blood FA concentrations in exposed persons [4]. Additionally, many rodent studies have indicated a correlation between a periconceptional high (2x or 10x) FA diet and negative physiological effects including behavioral and gene expression changes [[5], [6], [7], [8], [9], [10]]. Further, conflicting evidence in epidemiological studies published in 2018 has indicated investigation is needed to evaluate the possible correlation between diseases like autism spectrum disorders (ASD) and maternal FA overconsumption [[11], [12], [13]].

This study was done to determine if a 2x FA dose has an effect on gene expression and function of epigenetic modifying enzymes, histone modifications to Histone 3 (H3), DNA methylation, and dendritic spine densities in SHSY5Y cells. SHSY5Y cells have been used in a wide array of neurobiological studies and were used in this study [[14], [15], [16], [17]]. DNMTs, HMTs, HATs, HDACs, and other histone modifying enzymes such as ubiquitinases and phosphorylases were assayed for gene expression. Further, expression of MTFHR (methylene tetrahydrofolate reductase) was assayed since MTHFR is a crucial enzyme in conversion of FA to the methyl group. Activity of DNMTs, H3K4 HMTs, HDACs, and HATs as groups were measured by ELISA-based assays. Histone modifications to H3 and global DNA methylation were also measured by ELISA-based assays. Dendritic spines were counted as a measure of cellular changes since dendritic spines affect neuronal communication as spines are the site of glutamatergic synapses and therefore regulate excitatory neurotransmission. Data presented here indicate that a 2x FA dose in SHSY5Y cells affects gene expression of MBD2 and MECP2, the activity levels of DNMTs and H3K4 HMTs, levels of H3K4me1, H3K4me3, H3K9Ac, and global DNA methylation, and dendritic spine densities in SHSY5Y cells.

## 2 Materials and methods

### 2.1 Cell culture and treatments

SHSY5Y cells, a human neuroblastoma cell line, were chosen as the cell line for this study as they are frequently utilized in various types of neurobiological studies [[14], [15], [16], [17]]. All cell culturing supplies was purchased from Fisher Scientific. SHSY5Y cells (ATCC) were grown using DMEM:F12 supplemented with 10% fetal bovine serum and 1% penicillin/streptomycin in T75 tissue culture-treated flasks kept at 37 °C with 5% CO2 and high humidity. Upon reaching 80% confluence, cells were passaged using 0.05% trypsin-EDTA. Cells were not utilized for experiments if they were beyond passage 6. For experimentation, cells were plated in 6-well tissue culture treated plates at 200,000 cells/well.

Cells were treated a 2x FA dose 24 h post-plating. FA was purchased from Sigma. The DMEM:F12 medium contained 2.65 mg/L FA; therefore, the 2x FA cells received medium containing 5.3 mg/L FA in order to expose them to 2x the traditional FA amount. FA treatments lasted for 48 h before cells were harvested for further applications. This allowed for at least one doubling of cell number. There were at least 3 replicates per treatment group for each assay. The replicates were obtained from independent experimentation on different days. Details regarding sample acquisition (e.g. well numbers and plate numbers per sample) are described below in each section.

### 2.2 RNA isolation and gene expression analysis

For RNA experimentation, separate RNA samples were collected at different times on different days for the gene expression arrays and the Taqman assays. Each RNA sample included the contents of 4 wells from two 6-well cell culture plates; two wells from one plate and two wells from a separate plate to eliminate plate effects. RNA was isolated using the Purelink RNA Isolation Kit (ThermoFisher). RNA was quantified using a nanodrop.

For gene expression arrays, RT-PCR was performed to obtain cDNA using the RT2 First Strand Kit (Qiagen) to obtain cDNA; thermal cycling was done according to manufacturer instructions. QPCR was performed using epigenetic chromatin modifying enzyme array plates (Qiagen) and SYBR Green Mix (Qiagen). The plates consisted of primers for genes that encode chromatin modifying enzymes, including DNMTs, HATs, HMTs, HDACs, histone phosphorylases, histone ubiquitinases, and DNA/histone demethylases. A full list of genes in the array is provided in Table 1. The array plate gene groupings were derived from Qiagen's handbook supplied with the array plates.

**Table 1:** Columns are described here from left to right: In the left column, a list of genes, categorized by enzyme function that were on the qPCR array plate from Qiagen. The second column from the left has Refseq identifications for each mRNA assayed in the same order that gene names appear. In the third column from the left, gene functional group is provided. The fold change for genes with a significant uncorrected p-value is indicated in the third column from the right. The second column from the right has p values for genes with p < 0.05 using the paired two-tailed *t*-test. In the far right column are corrected p-values (using the Benjamini/Hochberg FDR) for genes that were p < 0.05 using the paired two-tailed *t*-test.

Gene Names	Refseqs	Function Group	Fold Change	P Value	Corrected P Value
KDM1A, KDM4A, KDM4C, KDM5B, KDM5C, KDM6B, MBD2	NM_015013, NM_014663, NM_015061, NM_006618, NM_004187, NM_001080424, NM_003927	Demethylase	MBD2: 1.20	MBD2: 0.00065	MBD2: 0.049
DNMT1, DNMT3A, DNMT3B	NM_001379, NM_022552, NM_006892	DNMT	DNMT1: 1.25	DNMT1: 0.0060	DNMT1: 0.225
ATF2, CDYL, CIITA, CSRP2BP, ESCO1, ESCO2, HAT1, KAT2A, KAT2B, KAT5, KAT6A, KAT6B, KAT7, KAT8, NCOA1, NCOA3, NCOA6	NM_001880, NM_004824, NM_000246, NM_020536, NM_052911, NM_001017420, NM_003642, NM_021078, NM_003884, NM_006388, NM_006388, NM_006766, NM_012330, NM_007067, NM_032188, NM_003743, NM_181659, NM_014071	HAT	KAT7: 1.34	KAT7: 0.04	KAT7: 0.311
HDAC1, HDAC2, HDAC3, HDAC4, HDAC5, HDAC6, HDAC7, HDAC8, HDAC9, HDAC10, HDAC11	NM_004964, NM_001527, NM_003883, NM_006037, NM_005474, NM_006044, NM_001098416, NM_018486, NM_178425, NM_032019, NM_024827	HDAC	HDAC4: 1.31 HDAC5: 1.28 HDAC6: 1.36 HDAC7: 1.49	HDAC4: 0.04 HDAC5: 0.04 HDAC6: 0.02 HDAC7: 0.04	HDAC4: 0.472 HDAC5: 0.299 HDAC6: 0.507 HDAC7: 0.413
CARM1, DOT1L, EHMT2, KMT2A, KMT2C, PRMT1, PRMT2, PRMT3, PRMT5, PRMT6, PRMT7, PRMT8, SETDB2, SMYD3, SUV39H1	NM_199141, NM_032482, NM_006709, NM_005933, NM_170606, NM_001536, NM_001535, NM_005788, NM_006109, NM_018137, NM_019023, NM_019854, NM_031915, NM_022743, NM_003173	HMT	DOT1L: 1.58 PRMT2: 1.35 PRMT5: 1.18	DOT1L: 0.03 PRMT2: 0.03 PRMT5: 0.04	DOT1L: 0.506 PRMT2: 0.513 PRMT5: 0.365
ASH1L, KMT2E, NSD1, SETD1A, SETD1B, SETD2, SETD3, SETD4, SETD5, SETD6, SETD7, SETD8, SETDB1, SUV420H1, WHSC1	NM_018489, NM_182931, NM_022455, NM_014712, NM_015048, NM_014159, NM_199123, NM_017438, NM_001080517, NM_024860, NM_030648, NM_020382, NM_012432, NM_016028, NM_007331	HMT Activity (SET Domain Proteins)	SETD4: 1.31	SETD4: 0.04	SETD4: 0.332
AURKA, AURKB, AURKC, NEK6, PAK1, RPS6KA3, RPS6KA5	NM_003600, NM_004217, NM_003160, NM_014397, NM_002576, NM_004586, NM_004755	Phosphorylase	NONE	NONE	NONE
DZIP3, MYSM1, RNF2, RNF20, UBE2A, UBE2B, USP16, USP21, USP22	NM_014648, NM_001085487, NM_007212, NM_019592, NM_003336, NM_003337, NM_006447, NM_012475, NM_015276	Ubiquitinase	NONE	NONE	NONE
ACTB, B2M, GAPDH, HPRT1, RPLP0	NM_001101, NM_004048, NM_002046, NM_000194, NM_001002,	Housekeeping Gene	N/A	N/A	N/A
HGDC	SA_00105	Genomic DNA	N/A	N/A	N/A

Each array plate contained the cDNA from one sample. Six array plates were used in total for six samples (three controls and three 2x FA-treated samples). The qPCR program described in the Qiagen booklet was utilized for incubation of each array plate. The array plates were analyzed by hand; the housekeeping (control) gene RPLP0 was chosen since its Ct values were the most similar for all array plates. Genomic DNA contamination wells were checked for a Ct value of >35 to ensure no genomic DNA contamination occurred. The ΔΔCt method was used to obtain relative abundance of each gene of interest. Relative abundance was compared across the control and 2x FA treatment groups using a paired two-tailed *t*-test followed by a Benjamini/Hochberg false discovery rate test to eliminate type-1 errors.

Two separate RNA sample sets were gathered during different months. These RNA samples were utilized for Taqman qPCR assays for DNMT1 (for validation/testing of DNMT1 array results), MECP2 (methyl CpG binding protein 2) and MTHFR (methylene tetrahydrofolate reductase). MECP2 was not on the array plate, but MECP2 is involved in epigenetic regulation of mRNA splicing [18] and maintaining a repressive methylated chromatin state [19]. MTHFR is involved in metabolizing FA to the methyl group that is donated to DNA and/or histones, and high FA consumption has been shown to decrease MTHFR expression in human hepatocytes [20].

CDNA was synthesized using the High Capacity cDNA Synthesis Kit (ThermoFisher). 500 ng of each RNA sample was input into each reaction. Thermal cycling for RT-PCR was done according to manufacturer instructions. FAM-labeled Taqman qPCR assays for DNMT1 (Assay ID: Hs00945875_m1), MECP2 (Assay ID: Hs00172845_m1), and MTHFR (Assay ID: Hs01114487_m1) were used to perform individual gene expression assays. The Taqman assay for the endogenous control, B-Actin (Accession Number NM_001101.2) was VIC labeled. QPCR was performed using Taqman Universal Mastermix (Applied Biosystems). The reaction mixes and cDNA samples were equally loaded into 96-well plates in quadruplicate. The qPCR reactions were incubated in a thermal cycler at 50 °C for 2 min, 95 °C for 10 min, and 40 cycles of 95 °C for 15 s and 60 °C for 1 min. The ΔΔCt method was used to obtain relative abundance of each gene of interest. Relative abundance was compared across treatment groups using paired two-tailed t-tests.

### 2.3 Nuclear extractions and Enzymatic Activity Assays

Nuclear extracts were obtained using a Nuclear Extraction Kit (Epigentek). Three samples were collected per treatment group during independent experiments on different days. Each sample consisted of the contents of 4 wells from 6-well tissue culture plates—2 wells from one plate and 2 wells from a separate plate constituted a single sample. Protein was quantified by Bradford assay using a plate reader. Enzymatic activity of DNMTs, HATs, HDACs, and H3K4 HMTs was measured using ELISA-based Enzymatic Activity Assays for each (Epigentek). Three replicates were measured in duplicate from each treatment group. Colorimetric results were read using a plate reader at 450 nm. Analyses were performed to determine enzyme activity according to Epigentek handbooks supplied with the kits. Activity of each enzyme category was compared across treatment groups using a paired two-tailed *t*-test.

### 2.4 DNA isolation and DNA methylation analysis

DNA was isolated using the PureLink Genomic DNA Mini Kit (ThermoFisher Scientific). Six samples were collected to obtain 3 control and 3 FA treatment samples. The contents of two wells were used to obtain one sample—one well from one 6 well plate and one well from a separate 6 well plate. DNA was quantified using a nanodrop. Global DNA methylation was measured using a MethylFlash Global DNA Methylation (5-mC) ELISA Kit from Epigentek. The DNA samples were loaded in duplicate into the ELISA plate along with four methylated DNA standards. The DNA samples were incubated with an antibody for 5-methyl Cytosine (5-mC) for 1 h followed by washes and incubation with a secondary antibody with a conjugated reporter. The plates were then washed again and a substrate was added followed by a solution to inhibit the reporter-substrate reaction. Plates were then read at 450 nm using a plate reader. The percentage of 5-mC in each DNA sample was calculated according to the instructions provided in the Epigentek manual. A paired two-tailed *t*-test was utilized to determine significance between the 3 control replicates and the 3 FA treatment replicates.

### 2.5 Histone isolations and histone modification analysis

Histones were isolated using a Histone Extraction Kit (Epigentek). Twelve wells constituted a single histone sample; therefore, two separate 6 well plates were harvested as a single sample. Six samples were obtained to have 3 control samples and 3 FA treated samples. The histone samples were quantified by Bradford assay. Equal amounts of histone were loaded into Histone 3 (H3) Multiplex Modification Array Kits (Epigentek). Each array plate contained 4 wells with antibodies for a specific histone modification to H3. Two wells per array plate were loaded with a control sample's histone extract in duplicate while the other two wells in that plate were loaded with a 2x FA sample's histone extract in duplicate. Histone modifications were bound to the wells by antibody. The wells were then washed and a secondary antibody with a conjugated reporter was added. The wells were washed again and a substrate was added followed by a solution to inhibit the reporter-substrate reaction. Plates were then read at 450 nm using a plate reader. A total of 3 array plates were used for 3 control samples and 3 FA treatment samples as each sample was assayed in duplicate. Analyses were performed according to the manual provided by Epigentek. The percentage of each modification was calculated as a function of the H3 modification level compared to the total H3 present in the sample. A paired-two tailed *t*-test was utilized to determine significance for each histone modification.

### 2.6 Cell staining and dendritic spine analysis

Cells were plated at 15,000 cells per coverslip on poly-d-lysine coated glass coverslips (Fisher). Three coverslips per treatment group were obtained during each round of the cell staining and spine analysis experiments; there were two independent rounds of experimentation on different days. After control or 2x FA treatment for 48 h, cells were fixed with 2% paraformaldehyde in PBS and were then stained with 1,1′-Dioctadecyl-3,3,3′,3′-Tetramethylindocarbocyanine Perchlorate dissolved in DMSO (“DiI” stain; Fisher Scientific) as previously described with modifications [21]. Cells were incubated in the dark at 37 °C for 2 h with warm DMEM:F12 media (with no FBS or antibiotic/antimycotic) with 5 μL/mL DiI and 4 μL/mL Lipofectamine reagent (Fisher). Cells were washed thrice with warm DMEM:F12 and were allowed to incubate overnight with DPBS at room temperature in the dark. The nuclei of the cells were counterstained with DAPI in DPBS the next morning. Coverslips were mounted on slides with Prolong Gold Antifade Mountant (Fisher) and were stored at 4 °C for two weeks before visualization and imaging with a confocal microscope using a 60× oil-immersion objective. Images were analyzed using ImageJ software (NIH). Dendritic spine density was determined as number of spines per 10 μm segment. One image was obtained randomly from each coverslip. Five counts were performed per image across a total of twelve coverslips per treatment group for a total of sixty counts. Spine densities were compared across treatment groups using a paired two-tailed *t*-test.

## 3 Results

### 3.1 Gene expression

**Fig. 1:**
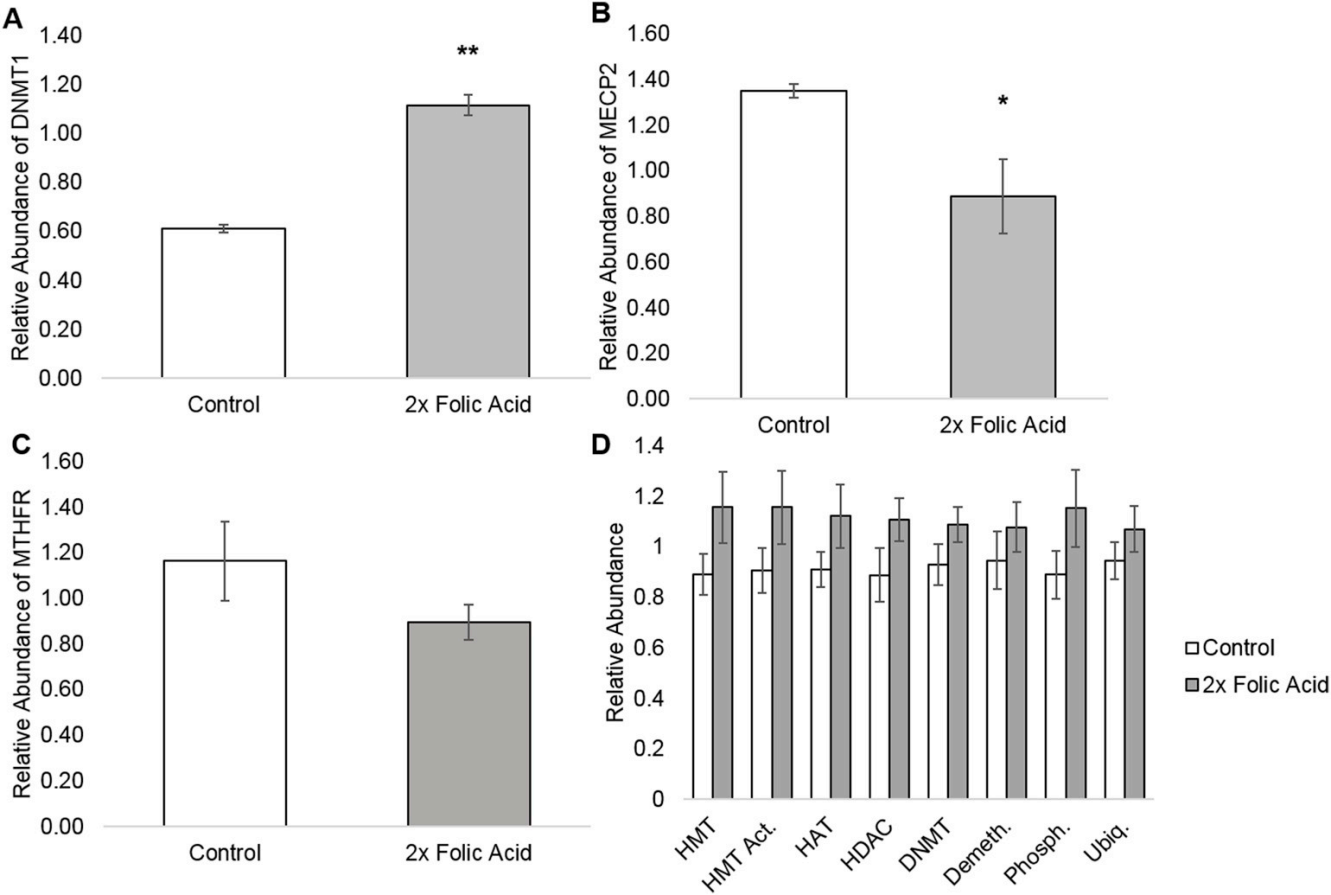
A. Relative abundance for DNMT1 mRNA expression measured by Taqman assay; p = 0.0038. B. Relative abundance for MECP2 mRNA expression measured by Taqman assay; p = 0.037. C. Relative abundance for MTHFR mRNA expression measured by Taqman assay; p = 0.14. D. Relative abundance of each gene grouping tested in the epigenetic chromatin modifying enzymes arrays. Gene groups tested were histone methyltransferases (HMTs, p = 0.058), HMT activity enzymes (HMT Act., p = 0.072), histone acetyltransferases (HATs, p = 0.074), histone deacetylases (HDACs, p = 0.061), DNA methyltransferases (DNMTs, p = 0.072), DNA/histone demethylases (Demeth., p = 0.17), histone phosphorylases (Phosph., p = 0.075), and histone ubiquitinases (Ubiq., p = 0.13). All Fig. 1 error bars are ± 1 standard error. A single asterisk (*) indicates p < 0.05, a double asterisk (**) indicates p < 0.01 using a paired two-tailed *t*-test.

After performing a paired two-tailed *t*-test, several genes including a few HDACs, HMTs, one phosphorylase, and one HAT had p < 0.05 (Table 1), but after correcting for type-1 errors, the only array gene with a significant change in expression was MBD2 (p = 0.047; Table 1). DNMT1 had the second lowest p-value, but after correction was not significant in the array. Upon studying DNMT1 expression further by Taqman assay, DNMT1 was significantly higher in the 2x FA group compared to the control (p < 0.01; paired two-tailed *t*-test; Fig. 1A). MECP2 was significantly decreased in the 2x FA group compared the control (p < 0.05; paired two-tailed *t*-test; Fig. 1B). MTHFR expression decreased in the 2x FA group, but the data was found to be insignificant (p = 0.10; *t*-test; Fig. 1C). After grouping the genes in the array by function, all function groupings’ p-values were higher than 0.05 (paired two-tailed *t*-test; Fig. 1D).

### 3.2 Enzymatic activity

**Fig. 2:**
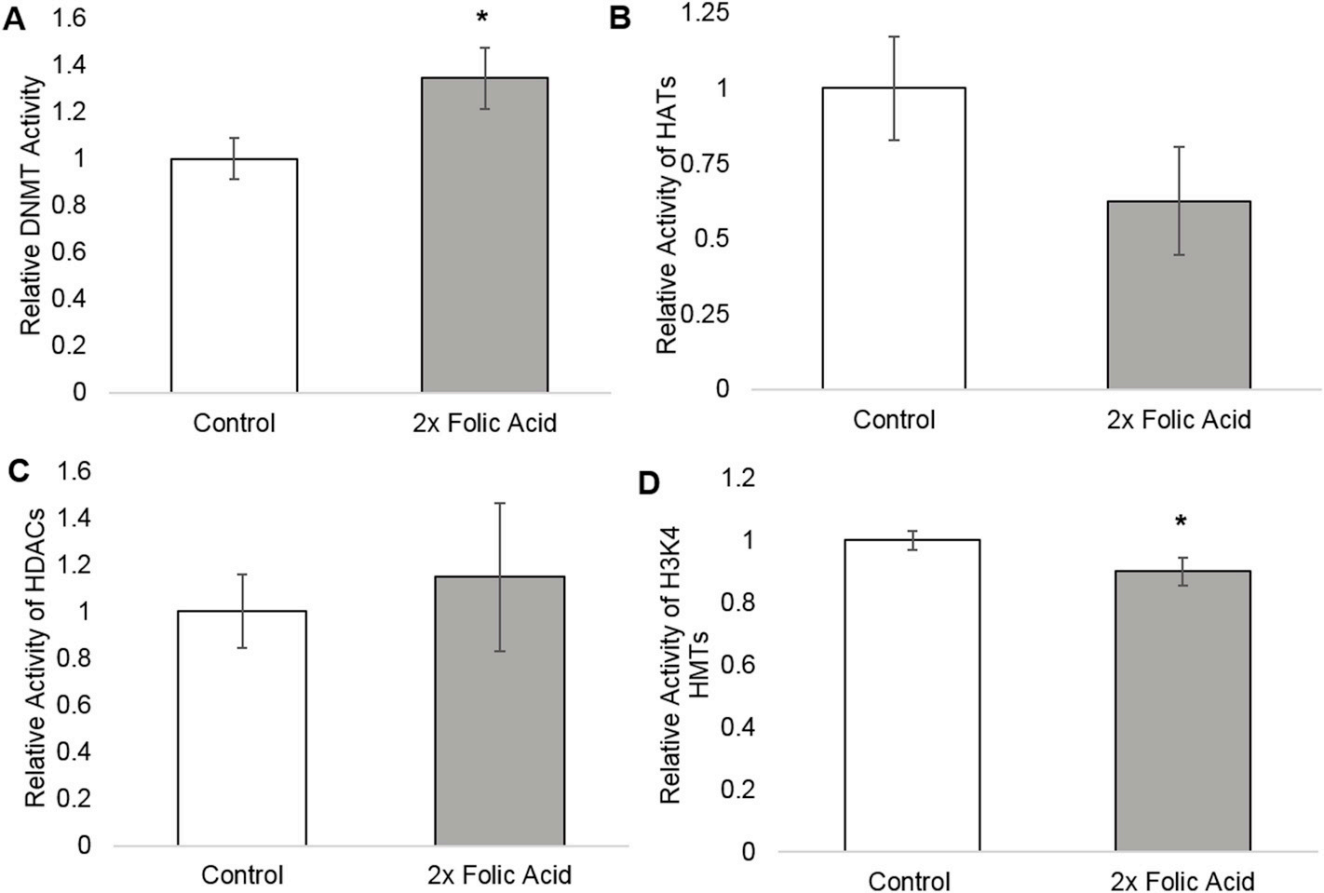
A. Relative DNMT Activity as measured by ELISA assay for control and 2x FA treated SHSY5Y cells; p = 0.046. B. Relative HAT Activity as measured by ELISA assay for control and 2x FA treated SHSY5Y cells; p = 0.084. C. Relative HDAC Activity as measured by ELISA assay for control and 2x FA treated SHSY5Y cells; p = 0.31. D. Relative H3K4 HMT Activity as measured by ELISA assay for control and 2x FA treated SHSY5Y cells; p = 0.048. For each assay, three replicates were tested per treatment group. All Fig. 2 error bars are ± 1 standard deviation. A single asterisk (*) indicates p < 0.05 using a paired two-tailed *t*-test.

DNMT activity was significantly increased in the 2x FA group (p < 0.05; paired two-tailed *t*-test; Fig. 2A). HAT activity decreased some in the 2x FA group, but the p-value was insignificant (p = 0.08; paired two-tailed *t*-test; Fig. 2B). HDAC activity increased some in the 2x FA group, but this was also insignificant (p = 0.10; paired two-tailed *t*-test; Fig. 2C). H3K4 HMT activity decreased significantly in the 2x FA group (p < 0.05; paired two-tailed *t*-test; Fig. 2D).

### 3.3 DNA methylation and histone modifications

**Fig. 3:**
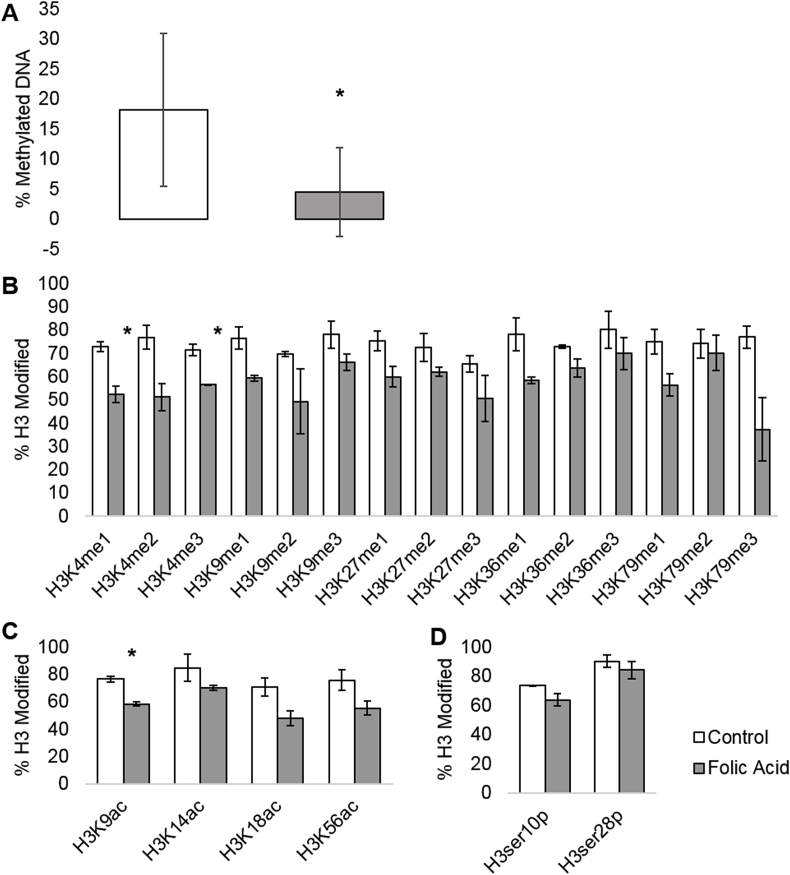
A. DNA methylation in SHSY5Y cells as determined by 5-mc ELISA assay; p = 0.048. Three replicates per treatment group were tested. Error bars are ± 1 standard deviation. B. Percentage of histone 3 (H3) modified with a particular methylation mark; p = 0.034 for H3K4me1, p = 0.073 for H3K4me2, p = 0.027 for H3K4me3, p = 0.065 for H3K9me1, p = 0.21 for H3K9me2, p = 0.18 for H3K9me3, p = 0.11 for H3K27me1, p = 0.18 for H3K27me2, p = 0.21 for H3K27me3, p = 0.094 for H3K36me1, p = 0.12 for H3K36me2, p = 0.28 for H3K36me3, p = 0.10 for H3K79me1, p = 0.40 for H3K79me2, and p = 0.10 for H3K79me3. Three replicates per treatment group were tested. Error bars are ± 1 standard deviation. C. Percentage of H3 modified with a particular acetylation mark; p = 0.018 for H3K9Ac, p = 0.21 for H3K14Ac, p = 0.10 for H3K18Ac, and p = 0.13 for H3K56Ac. Three replicates per treatment group were tested. Error bars are ± 1 standard deviation. D. Percentage of H3 modified with a particular phosphorylation mark; p = 0.13 for H3ser10p, and p = 0.31 for H3ser28p. Three replicates per treatment group were tested. Error bars are ± 1 standard deviation. An asterisk (*) indicates p < 0.05 using a paired two-tailed *t*-test.

DNA methylation was significantly decreased in the 2x FA treatment group compared to the control group (Fig. 3A). H3 lysine (K) methylation and acetylation were affected by the 2x FA treatment as well. There was a significant reduction in H3K4me1 (p < 0.05; *t*-test) and in H3K4me3 (p < 0.05; *t*-test) in the 2x FA treatment group compared to control (Fig. 3B). There were no other significant changes to H3 methylation. There was a significant reduction in H3K9ac in the 2x FA treatment group compared to control (p < 0.05; *t*-test; Fig. 3C). There were no other significant changes to H3 acetylation. There were no significant changes in serine phosphorylation of H3 between control and 2x FA treatment groups (Fig. 3D).

### 3.4 Dendritic spine density

**Fig. 4:**
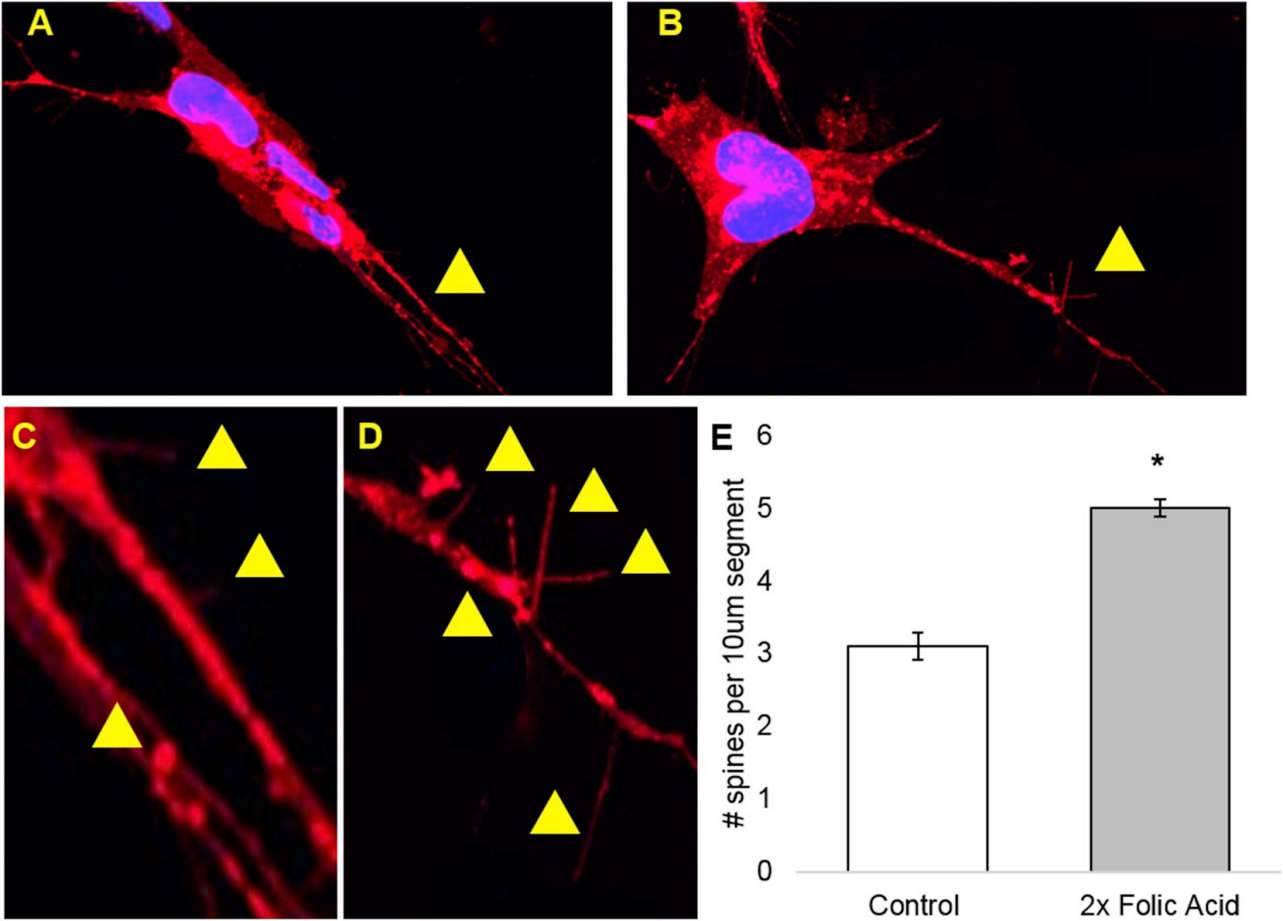
A and B. Representative images for control (A) and 2x FA treated (B) SHSY5Y cells. Images were used to perform dendritic spine counts. Yellow arrows indicate regions that are zoomed-in in C and D. C and D. Zoomed-in figures of representative images for control (C) and 2x FA treated (D) SHSY5Y cells. Yellow arrows indicate locations or regions of spines. E. Overall dendritic spine counts as number of dendritic spines per 10 μm segment; p = 0.00011. Error bars are ± 1 standard error. An asterisk (*) indicates p < 0.05 using a paired two-tailed *t*-test.

The 2x FA cells were notably more “hairy” in appearance when compared to the control group (Fig. 4A–D). Dendritic spine densities increased significantly in the 2x FA cells (p < 0.05; paired two-tailed *t*-test; Fig. 4E).

## 4 Discussion

This study began with the intent of determining if the 2x FA treatment would lead changes in gene expression and activity of chromatin modifying enzymes, histone modifications and global DNA methylation, and dendritic spine densities in SHSY5Y cells. The 2x FA treatment led to a significant decrease in expression of both MBD2 and MECP2 at the mRNA level. MBD2 and MECP2 were previously described as chromatin modifiers, each of which is significantly decreased in expression in ASD patients [[22], [23], [24], [25], [26]]. Decreased levels of MBD2 and MECP2 indicate less MBD2 and MECP2 could be available to bind methylated DNA to inhibit transcription. If so, a genome-wide analysis of mRNA levels could show increased expression of genes to which MBD2 and MECP2 are normally recruited.

Dendritic spine densities significantly increased in the 2x FA group. Increased dendritic spine density is associated with decreased MECP2 and is a feature of ASD [26]. An increase in dendritic spine density indicates decreased autophagy, a cell self-cleaning process, in the SHSY5Y cells [27]. An increase in spines also indicates an increase in excitatory neurotransmission. Mouse studies indicate high FA exposure results in higher anxiety-like behavior and hyperactivity [7] and increased repetitive behavior in exposed females [5]. High stress and anxiety are associated with increased repetitive behaviors [28] and decreased sociability [29]. Increased anxiety and repetitive behavior are primarily linked to abnormal activity and higher spine densities in the amygdala [30,31].

The changes to DNMT1 were not significant in the array results after correcting for type 1 errors. The Taqman assay for DNMT1 was performed on RNA samples gathered in a different month than the array RNA samples, and the Taqman assays were repeated once to reveal a significant increase in DNMT1 mRNA in the 2x FA group. DNMT activity was significantly increased in the 2x FA group, which may be reflective of the mRNA expression studies on DNMT1. Global DNA methylation, however, was significantly reduced in the 2x FA treatment group. This could be due to decreased MECP2, which is thought to assist with DNA and histone methylation [18].

HMTs that methylate H3K4 residues were significantly decreased in the 2x FA group. Interestingly, histone modification analysis revealed a significant decrease in H3K4me1 and H3K4me3 in the 2x FA group, which aligns with the H3K4 HMT activity analysis. H3K9Ac was also significantly reduced in the 2x FA treatment group even though activity of HATs and HDACs was not significantly altered. Other studies previously found that increases in H3K4me3 correlate with increases in H3K9Ac [3], which indicates an effect of histone methylation on HAT and/or HDAC expression or function [3]. Of note, H3K4me1, H3K4me3, and H3K9Ac were all significantly affected in the 2x FA group and are all features of ASD patients [19,20].

Upon grouping the array genes by function, the average relative abundances of the mRNA of each functional enzyme group were not significantly different between groups. HMTs, HMT Activity Enzymes, HATs, HDACs, and DNMTs had p values of 0.1 > p > 0.05 when comparing RNA expression between groups through paired two-tailed t-tests. HMT mRNA results likely do not align with H3K4 HMT activity results since the HMT mRNA grouping was for all HMTs regardless of their preferred methylation site. Oddly, insignificant changes to DNMT and HMT mRNA expression does not align with the significant changes to DNMT and H3K4 HMT activity, suggesting some post-transcriptional or post-translational modifications are at play.

The 2x FA treatment does lead to significant changes, some of which are observed in ASD patients. ASD-related changes include decreased MECP2 expression, decreased H3K4 methylation, decreased H3K9Ac, and increased dendritic spine counts [19,20,[22], [23], [24], [25], [26], [27]]. Though the employed 2x FA treatment to the SHSY5Y cells is by no means a physiological dose, we wanted to investigate the mechanistic effects of doubling the FA to which the SHSY5Y cells are normally exposed. Upon checking various suppliers, the recommended media for SHSY5Y cells (DMEM:F12) contains 2.65 mg/L FA, which is about 6  μM. Normal serum FA levels are, at maximum, 45.3 nM, which is roughly 100x less than the contents of the DMEM:F12, though much higher serum FA levels have been detected in various populations and are correlated with altered DNA methylation [32].

The obtained results occurred with only 48 h of 2x FA treatment, thereby only allowing one doubling of the cell line so that cells did not become overgrown while still supplying enough RNA, DNA, and histone proteins for study. Therefore, a longer treatment time could cause larger effects. This could especially be the case with MTHFR expression since MTHFR was not significantly altered, indicating there may be a lack of MTHFR's role in altered one-carbon metabolism in this experiment despite findings in previous experiments [20]. Further studies with longer treatment times could be useful in a cell line such as the SHSY5Y line that doubles about every 48 h. More, future studies would identify locations of DNA methylation changes through sequencing and further studies of genes' expression levels at sites of altered histone modifications (e.g. H3K4) through methods like ChIP-Seq. The findings presented here indicate it is possible too much FA could have detrimental effects in the cell. FA is crucial for survival, but it is suggested here that too much of an essential nutrient may be detrimental. Further studies are needed to determine if too much FA leads to harmful effects on the organism.
